# Convective Drying of Brown Seaweed (*Lessonia spicata*): Modeling, Energy Efficiency, and Impact on Bioactive Compounds and Functional Properties

**DOI:** 10.3390/foods14234011

**Published:** 2025-11-22

**Authors:** Sebastián Pizarro-Oteíza, Romina Cea, Millaray Aranda, Jéssica López, Erasmo Macaya, Fernando Salazar, Oscar Cavieres, Alejandra Sandoval-Bórquez, Roberto Lemus-Mondaca, Romina L. Abarca, Wladimir Silva-Vera

**Affiliations:** 1 Escuela de Alimentos, Pontificia Universidad Católica de Valparaíso, Av. Waddington 716, Valparaíso 2360100, Chile; 2 Laboratorio de Estudios Algales-AlgaLab, Departamento de Oceanografía, Universidad de Concepción, Cabina 3, Casilla 160-C, Concepción 4030000, Chile; 3 Laboratorio de Fermentaciones Industriales, Escuela de Alimentos, Pontificia Universidad Católica de Valparaíso, Av. Waddington 716, Valparaíso 2360100, Chile; 4 Escuela de Tecnología Médica, Facultad de Ciencias, Pontificia Universidad Católica de Valparaíso, Avenida Universidad 330, Curauma, Valparaíso 2373223, Chile; 5 Departamento de Ciencia de Alimentos y Tecnología Química, Universidad de Chile, St. Dr. Carlos Lorca 964, Independencia, Santiago 8380000, Chile; 6 Departamento de Ciencias Animales, Facultad de Agronomía y Sistemas Naturales, Pontificia Universidad Católica de Chile, Macul, Santiago 7820436, Chile; 7 Departamento de Biotecnología, Escuela de Industria Alimentaria y Biotecnología, Universidad Tecnológica Metropolitana, Las Palmeras 3360, Ñuñoa, Santiago 7800003, Chile

**Keywords:** brown seaweed drying, mathematical models, bioactive compounds, antioxidant capacity, energy requirement

## Abstract

Huiro negro (*Lessonia spicata*) is a brown alga with potential in the food, nutraceutical, and pharmaceutical industries. This study evaluated its convection drying at 30, 40, and 50 °C (2 ± 0.1 m/s; 83% RH), analyzing the kinetics using nine mathematical models. The Midilli–Kucuk model showed the best fit (R^2^ = 0.999). Drying at 50 °C significantly increased the fucoxanthin content (*p* ≤ 0.05) but had the highest energy cost (7.20 USD/kg). In contrast, 30 °C achieved the highest thermal efficiency (48.73%) and lowest cost (5.37 USD/kg), although with the lowest total phenolic compound (TPC) content. TPC was highest at 40 °C, while antioxidant capacity decreased to 30 °C and 40 °C, partially recovering at 50 °C. Higher temperatures also increased protein, fiber, and carbohydrates, while ash decreased. Tests on gastric cell lines (AGS and GES-1) showed low cytotoxicity at doses ≤ 1 mg/mL, with reductions in viability at concentrations ≥ 10 mg/mL. Overall, the extracts showed good biocompatibility at low doses and outstanding functional properties, especially after drying at 50 °C. The results show that the drying temperature significantly influences the nutritional and functional quality of *L. spicata*, favoring its use in functional foods.

## 1. Introduction

Globally, it is estimated that the population will increase from the current 8 billion people to approximately 10 billion in 2050, which implies an increased demand for food production. However, it is unlikely that this need can be met by land resources alone [1]. In this context, food resources from the ocean, such as algae, have been identified as one of the 50 foods of the future, with the potential to transform the global food system [2]. The use of algae as a source of new natural antioxidants and antibacterial substances, with a possible role as nutraceutical agents, can contribute to both food quality and food security [3].

Seaweed can be consumed in various preparations, mainly as vegetables in the diet; thus, the need arises to use different processing methods to preserve and improve the nutritional value to contribute to the adherence to consumption. From a nutritional point of view, algae are considered an underexploited source of protein, dietary fiber, polyunsaturated fatty acids, vitamins, and minerals [4,5]. Algae classification is according to their color: red (*Rhodophyta*), green (*Chlorophyta*), and brown (*Ochrophyta*, *Phaeophyceae*). All contain chlorophyll, although their pigmentation varies depending on their type. For example, green algae have chlorophyll a and b and carotenoids, similar to vascular plants. Macroalgae have chlorophyll a, phycobilins, and some carotenoids, while brown algae have chlorophylls a and c, in addition to carotenoids dominated by fucoxanthin, responsible for their brown color [6,7,8].

This food source is abundant and essential to coastal communities, as it offers great economic opportunities [9]. A prominent example is the genus *Lessonia*, especially *Lessonia spicata,* known as Huiro negro, a brown macroalga of economic importance in the food and pharmaceutical industries, whose wild harvest benefits many people [10]. In Chile, large volumes of algae are produced; according to FAO, 426,605 tons of algae were produced in 2019, highlighting the cultivation of 2 tons of *Macrocystis pyrifera* and 5 tons of red algae [11]. Brown algae have been identified as a renewable raw material with high added value, from which highly nutritious food products can be developed. Reformulating existing products and adding and replacing additives has the challenge of obtaining products that provide health-promoting nutritional properties, as reported by [12]. Additionally, these algae contain various bioactive compounds, such as polyphenols and carotenoids, which make them a raw material of great interest for food, nutraceutical, and pharmaceutical applications [13]. Polyphenols include phlorotannins, secondary metabolites exclusive to brown algae, derived from the polymerization of phloroglucinol, which have simpler structures than terrestrial plant tannins and are highly promising for the development of supplements and pharmaceuticals [14].

Another relevant compound within the carotenoids is fucoxanthin, a pigment of the xanthophyll family that gives the brown color characteristic of brown algae and possesses antioxidant and anti-inflammatory properties [15]. Also, brown algae exhibit higher levels of antioxidants compared to green and red algae, as reported by [16]. Their antioxidant activity is attributed to their ability to neutralize free radicals and reactive oxygen species and their metal-chelating ability, driven by compounds such as phenolic acids, catechin, caffeic acid, and quercetin [17].

Traditionally, the food industry has used brown seaweed for stabilizers and thickeners [18]. However, today they are gaining relevance as sources of nutrients and bioactive substances, especially their polysaccharides, which, although indigestible for humans, act as dietary fiber, induce satiety, improve intestinal function, and have beneficial effects on the microbiota [12].

On the other hand, one of the main problems of algae is their preservation because they contain a high percentage of moisture, around 90–95%, which makes them highly perishable [19]. In this sense, it is necessary to implement appropriate preservation methods in order to reduce the moisture content. Drying is presented as a viable solution, since it not only allows prolonging the shelf life of the algae but also contributes to their stability by reducing water activity, which generates the inactivation of the enzymatic system and inhibition of microbial growth [20]. A method commonly employed for this type of food is sun drying, which consists of directly exposing the algae to sunlight, either on the ground or on racks made of bamboo sticks and plastic sheets. This method can reduce the moisture content of the algae to approximately 40% [21]. However, drying in open spaces presents important limitations, such as contamination, rehydration of the product, and impairment of microbiological, biochemical, and sensory quality [22]. Because of these limitations, alternatives to solar drying have been developed, such as convective drying, which consists of applying an adequate amount of thermal energy through a flow of hot air, which facilitates the evaporation of the water contained in the food matrix [23,24,25].

Hot air drying stands out for its technical feasibility, simplicity, and lower energy cost compared to other methods of brown algae dehydration. Reference [26] demonstrated its efficiency in *Durvillaea antarctica*, showing good process control and moderate energy consumption. However, previous studies have focused mainly on kinetics or method comparisons, without integrating mathematical modeling, energy efficiency, and functional preservation. In this context, the analysis of convective drying of Huiro negro (*Lessonia spicata*) provides a comprehensive and applicable vision for the design of sustainable processes aimed at maintaining the nutritional and functional quality of local algae.

Hence, it is important to control the temperature during drying, as an excessive increase can promote organoleptic and nutritional changes, causing a degradation of product quality [27]. The time and temperature of the drying process are key variables that determine the quality of the final product. To determine these variables, mathematical models are used to predict the characteristics of the drying process, minimizing operational problems such as damage to the product, both in its physicochemical and nutritional properties, as well as excessive energy consumption [28]. These models, both empirical and semi-empirical, play a crucial role in estimating drying time, determining product moisture content, and adjusting process parameters, thus contributing to greater energy efficiency and preservation of product quality [29]. Thus, it is essential to consider the operating parameters as they influence the effective diffusivity calculations, which allow modeling drying kinetics that impact process efficiency [26,30,31]. Mathematical models are essential tools for evaluating the influence of factors such as temperature and material thickness on the drying rate, particularly in products arranged in thin layers [27,32].

Various empirical and semi-empirical models, including the Page, modified Page, Weibull, Wang and Singh, Henderson and Pabis, Midilli–Kucuk, logarithmic, and Verma models, have been applied to describe moisture diffusion and predict drying kinetics in brown algae [26]. Accurate estimation of the energy demand associated with drying operations is also crucial to ensure process efficiency and sustainability, as water removal through evaporation is inherently energy-intensive [33]. Energy consumption not only influences operating costs but also determines the long-term economic and environmental feasibility of the process [34].

Therefore, this study aimed to evaluate the convective drying of the brown macroalga *Lessonia spicata* by modeling its drying behavior, assessing energy efficiency, and determining the effects of the process on bioactive compounds and functional properties.

## 2. Materials and Methods

### 2.1. Raw Materials

Huiro negro (*Lessonia spicata*) was collected from Caleta El Membrillo de Valpara-íso, Region of Valparaíso, Chile. The raw material was washed to remove excess salt and other contaminants. Whole seaweed underwent blanching to inactivate degradative enzymes and prevent the formation of volatile compounds responsible for rotting odors.

In addition, samples of Huiro negro were collected at two key stages of its annual growth: in July and November, thus obtaining two different batches or blocks for all the experiments evaluated. The first batch weighed 15 kg and the other 14.2 kg.

**Figure 1:**
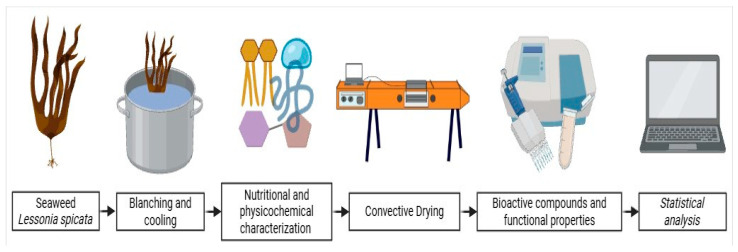
Scheme of methodology performed.

Blanching was performed for 7 min at 95 °C, following the protocols described by [35,36], based on the experiment by [37]. After blanching, the algae were manually cut with a stainless-steel knife into rectangular pieces measuring approximately 3 cm × 0.5 cm × 0.2 cm, and then rapidly cooled and stored at −18 °C until further analysis [31]. The complete methodological scheme is presented in Figure 1.

### 2.2. Nutritional and Physicochemical Characterization

Seaweed samples were analyzed for total protein, carbohydrate, lipid, ash, and dietary fiber content. Moisture and ash content were determined using official AOAC (2005) methods, consistent with those applied by [38] for food samples.

Specifically, samples were dried in an oven at 105 °C for 24 h, and ash was obtained by incineration at 550 °C for 12 h. Total carbohydrate content was quantified using the phenol-sulfuric acid method described by [39]. Crude protein was determined using the Kjeldahl nitrogen assay [40].

Total fiber content was analyzed using the AOAC enzymatic-gravimetric method [41]. Lipid content was determined by extraction in a Soxhlet apparatus, following the procedure described by [42]. All analyses were conducted in triplicate, and results are reported as means with standard deviations (*n* = 3) on a dry basis. Water activity (aw) was measured by placing samples in a WP-40TH (Rotronic, Bassersdorf, Switzerland) water jacket sample holder equipped with an HC2-AW capacitive measuring probe connected to a portable reader (HP23-AW-A), providing aw values with an accuracy of ±0.01. The water jacket sample holder was maintained at a constant temperature using a Lauda Alpha A6 thermal bath.

### 2.3. Convective Drying Procedure

Samples were thawed for 24 h at 3.0 ± 0.1 °C. A 150 g sample was weighed and placed on a grid tray for drying in a convective hot air tunnel (UOP 8 Tray Dryer, Armfield, Ringwood, UK) as shown in Figure 1. Drying was conducted at temperatures of 30, 40, and 50 °C with an air velocity of 2.0 ± 0.1 m/s [31], measured using a digital anemometer (VWR International Ltd, Dublín, Ireland). Relative humidity (RH) was monitored by a hygrometer (MA-45, Sartorius AG, 37075 Göttingen, Germany) and a value of 83% was obtained. Weight loss was measured at de-fined intervals using a digital analytical balance (YMC Co., Ltd., Kyoto, Japan) connected to a computer interface system, which recorded and stored data until a constant weight was achieved.

#### Drying Kinetics, Drying Rate, and Empirical Models

Drying kinetics were recorded at 7 min intervals. Mass loss was measured using a balance until equilibrium moisture content was achieved. Data collected at three temperatures (30, 40, and 50 °C) were plotted as the moisture loss rate (MR, dimensionless) over time and fitted to eight empirical models: Page, Weibull, Wang and Singh, Henderson and Pabis, Midilli–Kucuk, Logarithmic, Verma, and Verma modified (see Table 1). The moisture ratio (MR) was calculated using Equation (1), and the drying rate (DR) was determined by Equation (2) [43].

(1) $$MR=\frac{W_{t}-W_{e}}{W_{o}-W_{e}}$$

(2) $$DR=\frac{W_{t2}-W_{t1}}{t_{2}-t_{1}}$$

where W_t_ (g water/g dry matter) is the moisture content at time t, W_e_ (g water/g dry matter) is the equilibrium moisture content, which was assumed to be negligible [44] and W_o_ (g water/g dry matter) is the initial moisture content, W_t1_ and W_t2_ are the moisture content on a dry basis at times t_1_ and t_2_, respectively.

### 2.4. Estimation of the Effective Diffusion Coefficient (D_eff_)

To evaluate the effective moisture diffusion of seaweed samples, it was supposed slab geometry during drying, leading to use the simplified equation of Fick’s law for moisture diffusion [51] (Egbe, 2023), which is represented as follows:

(3) $$MR=\frac{M}{M_{o}}=\frac{8}{\Pi^{2}}\sum_{n=1}^{\infty }\frac{1}{{\left(2n-1\right)}^{2}}exp\left(-\frac{{\left(2n-1\right)}^{2}\Pi^{2}D_{eff}t}{4L^{2}}\right)$$

where D_eff_ is the effective moisture diffusivity of the material (m^2^/s), L is half the thickness of the algae (m), t is the drying time (s), and n is taken as 1 for longer drying times. The most widely studied theoretical model for thin film food drying is derived from the solution to Fick’s second law, which was applied to fit the experimental drying data. For sufficiently long drying times, Equation (3) is expressed in logarithmic form as follows:

(4) $$ln(MR)=ln\left(\frac{8}{\Pi^{2}}\right)-\frac{\Pi^{2}D_{eff}t}{4L^{2}}$$

The slope method was employed to determine the effective moisture diffusivity coef-ficient (D_eff_). Experimental drying data were plotted as ln (MR) versus drying time (t), al-lowing for the calculation of D_eff_ [52]. As shown in Equation (5), the plot produces a straight line with a slope (k), from which the D_eff_:

(5) $$k=\frac{\left(\Pi^{2}D_{eff}\right)}{4L^{2}}$$

The activation energy (Ea) was determined from the mass diffusion coefficient based on Arrhenius-type dependence. The influence of drying temperature on kinetic processes was evaluated using the Arrhenius Equation (6) [53].

(6) $$D_{eff}=D_{o}exp\left(\frac{-E_{a}}{RT}\right)$$

where E_a_ is the activation energy (J/mol), R is the universal gas constant (8.3123 J/mol/K), T is the absolute air temperature (K) and D_o_ indicates the base of diffusion in the material (m^2^/s). After linearization, the slope indicates the activation energy. Equation (7), according to [51], the plot of the natural logarithm of D_eff_ versus the inverse of the absolute temperature produces a straight line whose slope is K_1_, from which the following is calculated of activation energy (E_a_).

(7) $$k_{1}=\frac{E_{a}}{R}$$

### 2.5. Energy Requirement

A digital electric counter (UT210E, Unit Trend Technology, Dongguan, China) was used to measure the voltage and current consumption through the drying process. The dry bulb (T_db_) and wet bulb (T_wb_) air temperatures were continuously monitored during dehydration [54]. Energy balances were applied to both air-heating and water evaporation steps in the dehydration process, as follows:

Energy absorbed by air

(8) $$E_{Abs.-Air}={\dot{m}}_{air}{\bar{Cp}}_{air}\left(T_{P}-T_{inlet}\right)t=\bar{v}A\rho_{air}{\bar{Cp}}_{air}\left(T_{P}-T_{inlet}\right)t$$

Energy absorbed by water

(9) $$E_{Abs.-Water}[kWh]={\dot{M}}_{water-vap.}\lambda_{\left(T\right)}t=M_{drysolid}\sum_{t=0}^{t=t}{\left[\frac{∆X_{d.b}}{∆t}\right]}_{t}\lambda_{\left(T\right)}t$$

In Equation (8), $E_{Abs.-Air}$ is the energy absorbed by air (kWh), ${\dot{m}}_{air}$ is the mass flow rate of air (kg/s) calculated by $\bar{v}A\rho_{air}$, $\bar{v}$ is the average air velocity (m/s), Flow area (m^2^) and air density (kg/m^3^) respectively, ${\bar{Cp}}_{air}$ is the specific heat capacity of air (kJ/kg/K), $T_{P}-T_{inlet}$ is the difference between processing temperature and air inlet temperature (K) and t is time (s).

On the other hand, in Equation (9), $E_{Abs.-Water}$ is the energy absorbed by water (kWh), ${\dot{M}}_{water-vap.}$ is the mass of water vaporized (kg water), $\lambda_{\left(T\right)}$ is the latent heat of evaporation of water at temperature T (kJ/kg water), $X_{d.b}$ is the dry-basis moisture content (kg_water_/kg dry matter), and t is time (s).

The Overall Thermal Efficiency (OTE) ($\eta_{Overall}$) for *L. spicata* dehydration was determined using Equation (11), as described by [55]. In this study, only electrical energy heaters were considered as total energy consumed (E_TOTAL_) [kWh] as follows:

(10) $$E_{TOTAL}=V\cdot I\cdot t$$

where V is the voltage (V), I is the current (A), and t is time (h).

(11) $$\eta_{Overall}=\eta_{Air\;Heating}\cdot \eta_{Water\;Remotion}=\frac{E_{Abs.-Air}}{E_{Total}}\cdot \frac{E_{Abs.-Water}}{E_{Abs.-Air}}$$

where $\eta_{Air\;Heating}$ is the thermal efficiency for air heating, $\eta_{Water\;Remotion}$ is the thermal efficiency for water remotion and $E_{Total}$ is the total energy consumed (kWh).

#### 2.5.1. Specific Energy Consumption (SEC)

Efficiency in energy consumption during the *L. spicata* drying process was evaluated from Specific Energy Consumption (SEC) [kWh/kg water] [56].

The SEC value represents the total energy consumption during drying per mass of vaporized water.

(12) $$SEC=\frac{E_{TOTAL}}{M_{water-vap.}}$$

#### 2.5.2. Overall Mass Transfer Coefficient (K_G_)

The mass transfer coefficient (KG) was calculated for each drying step using Equation (13). This coefficient represents both water diffusion within the matrix and water-vapor diffusion across the boundary layer between the solid and gas phases [57]. The following analysis examines the effect of air processing temperature on KG:

(13) $$M_{dry\;solid}\sum_{t=0}^{t=t}{\left[\frac{∆X_{d.b}}{∆t}\right]}_{t}\lambda_{\left(T\right)}=\lambda_{\left(T_{wb}\right)}K_{G}A_{total\;tray}\left(P_{sat.}-P_{Vap.}\right)$$

where P_sat_. is the saturated vapor pressure (Pa), P_vap_. is the vapor pressure (Pa), λ_(Twb)_ is the latent heat at T_wb_ (kJ/kg water), and A_Total tray_ is the total tray area (m^2^). P_sat_ was estimated from the Tetens equation at T_wb_ and P_vap_ was related to temperature, as shown by [58].

### 2.6. Bioactive Properties

#### 2.6.1. Total Phenolic Content (TPC)

Phenolic compounds and flavonoids were extracted from brown seaweed L. spicata following the procedure described by [59], with minor modifications. A 2 g sample was weighed and extracted with 20 mL of 60% methanol. Extraction was conducted at room temperature (29 ± 2 °C) for 24 h in the dark at 100 rpm using an incubator and shaker. The total phenolic content (TPC) was determined according to the method of [60], with modifications. In a microplate, 75 µL of water, 20 µL of 1N Folin–Ciocalteu reagent, and 10 µL of Huiro negro extract were combined and allowed to stand for 5 min.

Then, 30 µL of saturated Na_2_CO_3_ solution (10%, *w*/*v*) and 120 µL of distilled water were added. The mixture was incubated in the dark at room temperature for 2 h. Absorbance was measured at 760 nm and compared with standards contain-ing known concentrations of phenolic compounds. TPC values were expressed as mg gallic acid equivalents (GAE) per 100 g dry weight.

#### 2.6.2. Total Flavonoid Content (TFC)

The total flavonoid content (TFC) of the extracts was determined according to the method described by [61], with minor modifications. Quercetin served as the standard for the calibration curve. Extracts were diluted at a factor of 10. In a microplate, 75 µL of methanol, 5 µL of 10% *w*/*v* aluminum chloride, and 25 µL of the sample were sequentially added and allowed to stand for 3 min. Subsequently, 5 µL of 1M potassium acetate and 140 µL of distilled water were introduced. After incubation at room temperature for 30 min, the absorbance was measured at 415 nm using a spectrophotometer. TFC was reported as milligrams of quercetin equivalents (QE) per 100 g dry weight.

#### 2.6.3. Carotenoid Total and Fucoxanthin Content

Carotenoids (µg/mL) were extracted using 100% methanol by mixing 10 mL of methanol with 0.1 g of brown algae. For fucoxanthin extraction, the method described by [62] was applied with modifications, utilizing an acetone-water mixture (4:1, *v*/*v*) with 10 mL of solvent and 0.1 g of brown algae. Both mixtures were vortexed for 1 min at room temperature while shielding samples from light. The resulting ex-tracts were centrifuged at 2500 RPM for 10 min to collect the supernatant, which was then subjected to a second centrifugation at 5000 RPM for 5 min.

(14) CT=4α

(15) FC=7.69α−5.55β−0.377γ

where α = $\left(A_{480}-A_{750}\right)$, β = $\left[\left(A_{631}-A_{750}\right)+\left(A_{582}-A_{750}\right)-0.297\left(A_{665}-A_{750}\right)\right]$, and γ = $\left(A_{665}-A_{750}\right)$.

#### 2.6.4. Antioxidant Capacity (AC)

The DPPH radical scavenging assay was performed as described by [63]. In this procedure, 100 µL of the sample and 100 µL of DPPH methanolic solution were added to each well of a microplate. The mixture was incubated in the dark at room temperature for 30 min. Absorbance was measured at 517 nm. The percentage inhibition was determined using Equation (16):

(16) $$\%DPPH\;Inhibition=\left(\frac{Abs\;control-Abs\;sample}{Abs\;control}\right)100$$

#### 2.6.5. Cell Viability Assay

The AGS human cell line was obtained from the American Type Culture Collection (ATCC, Manassas, VA, USA), and the GES-1 human cell line was kindly provided by Dr. Armando Rojas (Universidad Católica del Maule, Talca, Chile).

GES-1 (normal gastric epithelium) and AGS (gastric adenocarcinoma) cell lines were maintained in RPMI 1640 medium supplemented with 10% FBS, penicillin (100 U/mL), and streptomycin (100 µg/mL) at 37 °C, 5% CO_2_. Cells (2.0 × 10^4^/well) were seeded in 48-well plates and after 24 h, treated with fresh or 50 °C-dried *Lessonia spicata* extracts (0.1–50 mg/mL) or vehicle (60% methanol) for 24 h.

Viability was assessed by trypan blue exclusion in a Neubauer chamber, as previously described by [64]. Statistical analysis: Welch’s ANOVA with Dunnett’s T3 post hoc; α = 0.05.

### 2.7. Statistical Analysis

Experiments were conducted in triplicate; as for the statistical parameters used as criteria to determine the model that best fit the moisture rate-time relationship data, they were the coefficient of determination (R^2^) [65], the sum of squared errors (SSE; Equation (17)) [66] the Root Mean Square Error (RMSE; Equation (18)) [67], the chi-square (χ^2^; Equation (19)) [68] and the Akaike Information Criterion with bias correction (AICc) [69].

(17) $$SSE=\frac{1}{N}\sum_{i=1}^{n}{\left({MR}_{exp}-{MR}_{pred}\right)}^{2}$$

(18) $$RMSE=\sqrt{\frac{1}{N}\sum_{i=1}^{n}{\left({MR}_{exp}-{MR}_{pred}\right)}^{2}}$$

(19) $$\chi^{2}=\frac{\sum_{i=1}^{n}{\left({Exp}_{i}-{Cal}_{i}\right)}^{2}}{N-z}$$

(20) AIC=2p−2Ln(L)

(21) $$Ln(L)=0.5\left[-N_{res}\left(Ln\left(2\pi \right)+1-Ln\left(N_{res}\right)+Ln\sum_{i=1}^{n}x_{i}^{2}\right)\right]$$

(22) $$AICc=AIC+\frac{2p\left(p+1\right)}{n_{res}-p-1}$$

where i represent a specific experiment, N is the total number of experiments, ${Exp}_{i}$ refers to experimental values, ${Cal}_{i}$ represents the calculated values, y Exp is the average of the experimental values, z is the number of model constants, p is number of parameters, Ln(L) is the maximum log-likelihood of the estimated model, x_i_ is the residual from the non-linear least-square fit, N_res_ is number of residual, n_res_ is number of residual sample, and ${MR}_{exp}$ y ${MR}_{pred}$ is the experimental and predetermined moisture ratio, respectively. Analysis of variance (ANOVA) was conducted using Statgraphics Centurion v. 19.7.02 statistical software at a 95% confidence level. Duncan’s multiple range tests were subsequently applied to the data for total phenolic content (TPC), total flavonoid content (TFC), carotenoids total (CT), Fucoxanthin content (FC), percentage inhibition (DPPH), and proximate analysis of both fresh and de-hydrated seaweed samples. The results were expressed as mean values ± standard deviation.

## 3. Results and Discussion

### 3.1. Nutritional and Physicochemical Composition

Table 2 presents the values of the proximate composition of *Lessonia spicata*. These values can be influenced by various factors that affect biomass conditions, such as species, developmental stage, geographic distribution, season of the year, exposure to waves and ocean currents, nutrient concentration, salinity, water depth, and temperature [70].

Huiro negro being a brown macroalgae, the conformation of its tissues facilitates water uptake to maintain hydration and osmotic balance [71]. Also, their cell walls contain hydrophilic polysaccharides, such as alginate, which has a high water-holding capacity [72]. It was observed that the different moisture values obtained at drying conditions of 30, 40, and 50 °C were statistically different from each other (*p* < 0.05). They also present a statistical difference with the moisture value of the fresh sample, 87.46 ± 0.03 g of water/100 g of dry basis (*p* < 0.05). This high moisture content makes them highly perishable, requiring immediate consumption or processing to avoid deterioration or microbial growth [73]. This value is comparable to that reported for other brown algae such as *Ulva intestinalis* (84.34 ± 0.48 g of water/100 g of wet basis) and decreases significantly with drying as the temperature increases [23].

Water activity is an indicator of the stability of a food, which, as it decreases, presents less risk of microbial contamination, generally concentrating the solid components, which contributes to the preservation of the quality and safety of the product [74]. In this study, a significant reduction in this parameter was observed with the convective drying process as the temperature increases. For the fresh sample, the value obtained was 0.98 ± 0.01, while for the dehydrated samples at 30, 40, and 50 °C, significantly lower values of 0.48 ± 0.00, 0.32 ± 0.01, and 0.25 ± 0.01, respectively, were obtained (*p* < 0.05). According to [75], microbial growth is promoted as water activity increases but cannot occur when a_w_ < 0.6, which underlines the importance of achieving low values by dehydration processes. Similarly, the development of microorganisms and the action of endogenous enzymes present in the algae were prevented by prior blanching of the food matrix, a process that reduces the thermolabile microbial load and inactivates enzymes that cause enzymatic browning or degradation [35].

Regarding lipids, significant differences were observed according to drying temperature, reaching a higher value at 40 °C (1.94 ± 0.09 g/100 g dry matter).

These results are consistent with previous studies reporting low lipid levels in brown macroalgae (1–5% dry matter) [76]. Although the amount of lipids in algae is limited, they stand out for their content of polyunsaturated fatty acids, such as omega-3 fatty acids: α-linolenic acid, eicosapentaenoic acid, docosapentaenoic acid, and docosahexaenoic acid. Species such as *Chlorella vulgaris* and *Arthrospira platensis* have been used to develop extracts that can be used as dietary supplements, recognized for their biological benefits [77]. However, not all lipids have favorable physiological effects. Therefore, it is essential to characterize the fatty acid composition in different brown algal samples to better assess their potential applications [78].

Lipid composition can vary according to factors such as species, season, and processing method [78]. For example, it has been reported that in other algae, such as green *E. clathrata*, lipid content reaches 4.6%, followed by the red alga *Gracilaria folifera* with 3.23%, and brown algae such as *Codium tomentosum* with 2.53% [79]. These findings highlight the importance of studying and differentiating lipid properties among different types of macroalgae. On the other hand, protein content increased progressively with drying temperature, showing significant differences, reaching a value of 14.16 ± 0.35 g/100 g dry matter at 50 °C. This increase is attributed to the concentration of solid components after evaporation of water during the process [74]. Although the Kjeldahl method is a widely used technique for estimating protein content in algae, its accuracy can be affected by variability in nitrogen-protein conversion factors. This method assumes that most of the nitrogen present in the sample comes from protein, which can lead to an overestimation [80]. In addition, since there is no universally established conversion factor, the calculated protein values may vary depending on the environmental conditions at the sampling sites or the factors used [81].

In relation to ash, a significant decrease was observed with drying, going from 14.48 ± 0.01 g/100 g dry matter in the fresh sample to 12.94 ± 0.01 g/100 g dry matter at 50 °C. This decrease indicates a higher mineral content in the sample, which positions *L. spicata* as a dietary supplement. However, high levels of ash (>10%) may limit its use in food, since, in poultry diets, for example, it may negatively affect digestion due to possible interference with the absorption of other nutrients [5]. In comparison with other Chilean species, the values obtained in this study for the fresh sample were close, since in brown algae, ash values between 14% and 45% have been reported [82]. The variability of ash content reflects the differential ability of species to accumulate inorganic elements from seawater, which may depend on internal factors such as the type of polysaccharides in the cell wall and the age of the plant. According to [83], the ash content in macroalgae can reach up to 40% of its dry matter, although in this case the values found are lower. On the other hand, ref. [84] indicated that the drying method does not present significant differences in the ash content of macroalgae, but some studies report higher values. For example, the ash content of *Laminaria japonica* was 64.3%, which exceeds the values found in this study for *L. spicata.* This fact could be related to the method of storage chosen for *L. japonica,* as prolonged immersion in saltwater could promote further sodium accumulation in the algae [84]. In addition to the different harvest seasons in which the samples were obtained in our study, it is possible that these may have influenced the initial moisture content, which, in turn, modifies the concentration of solid matter in the samples. Regarding the total dietary fiber content, the fresh sample presented values of 8.97 ± 0.48 g/100 g dry matter, while at 50 °C it reached 62.35 ± 0.48 g/100 g dry matter, showing a significant difference as a function of drying temperatures.

The highest content was recorded at the highest drying temperature, which is due to the reduction in water during the process, which concentrates the solid components such as polysaccharides present in the algae, mainly alginates. Generally, dietary fiber in algae can range from 5 to 69% of dry matter, and alginate polysaccharides are resistant to degradation by endogenous enzymes in the digestive tract but can be broken down and fermented by the gut microbiota, promoting beneficial health effects [78]. Therefore, brown macroalgae can be considered an excellent source of dietary fiber and have been widely used in food applications because of their potential. The values obtained in this study are higher than those reported in the literature for various species of algae, such as *Sargassum muticum*, which presented values of 5.5–10.1 g/100 g [76]. These variations are related to structural adaptations to the hydrodynamic stress of each species. Finally, several studies have reported that carbohydrates from brown algae contain higher levels of fiber than those found in common fruits and vegetables [85]. In the case of the brown macroalga *L. spicata*, the content of available carbohydrates was lower than that of fiber, and no significant differences were observed between the fresh sample and the one dried at the higher temperature (*p* < 0.05). However, as shown in Table 2, the highest available carbohydrate content was reached at 50 °C, with a value of 61.27 ± 0.31 g/100 g dry matter. This behavior can be explained by the mannitol present in dehydrated brown algae such as *Laminaria digitata* and *Ascophyllum nodosum, which* represents between 20 and 30% of the dry matter [86]. Although brown algae contain carbohydrates such as mannitol and other simple sugars, their concentration is usually lower compared to structural polysaccharides such as alginates. These simple carbohydrates represent only a fraction of the total dry matter [86]. In the case of the study alga, alginic acid constitutes a large portion of total carbohydrates, indicating that polysaccharides are present in dietary fiber [87].

### 3.2. Drying Kinetics, Mathematical Modeling, and Drying Rates

The data obtained for the three temperatures were fitted to eight empirical kinetic models, which are presented in Table 1. Higher drying temperatures accelerated the exponential decrease in moisture content, significantly reducing drying time (*p* < 0.05) due to enhanced mass transfer rates [88]. However, ref. [89] noted that temperatures above 50 °C offer limited additional benefit for shortening the process.

The parameters calculated using the different models together with their adjustment statistics are shown in Table 3. Comparing the parameters obtained for the Page model, “k” tends to increase as the temperature increases. The same happens with the Henderson and Pabis model (0.0257 at 30 °C, 0.070 at 40 °C, and 0.0732 at 50 °C), indicating faster drying [90]. In the case of the Weibull model, it delivers the parameters α and β, where α corresponds to a scale parameter and β is a shape parameter. As the temperature increases, the value of β decreases, indicating a different drying pattern or a wider drying time distribution.

In general, it is observed that most of the models related to the drying rate (“k” value) tend to increase as the drying temperature increases, accelerating drying.

Regarding the goodness-of-fit statistics, the R^2^, which represents the proportion of the variance in the dependent variable that is predictable from the independent variable, where a higher fit is related to a value close to 1 [65]. At 30 °C all the models present very high values of fit (>0.995), where the lowest value was obtained by the Henderson and Pabis model. The same happens with 40 °C temperature, where the model obtained an R^2^ value of 0.938, with a tendency of decreasing values in all models. At 50 °C, the Verma and Midili-Kucuk models obtained the highest R^2^ values, meanwhile the Henderson and Pabis model presented the lowest R^2^ (0.975).

The Midilli–Kucuk model showed the best fit with respect to R^2^ (0.999) regardless of air-drying temperature. The sum of squares error (SSE) measures the total deviation of the predicted values from the experimental values; as the SSE value decreases, it indicates a better fit [66]. At 30 °C, the models presented low values (0.001 to 1.077), being the Midilli–Kucuk model and the Henderson and Pabis model with lower and higher fit, respectively. In addition, at 40 °C and 50 °C, it is observed that the Midilli–Kucuk, Verma, and Verma modified models present the lowest SSE, indicating a good fit of this model.

The chi-square statistic (χ^2^), which measures the difference between the observed and expected values, as the values increase, there is a lower fit. At 30 °C, the models present a range of χ^2^ of 0.00004–1.137, whereas at 40 °C and 50 °C, they were slightly higher.

**Figure 2:**
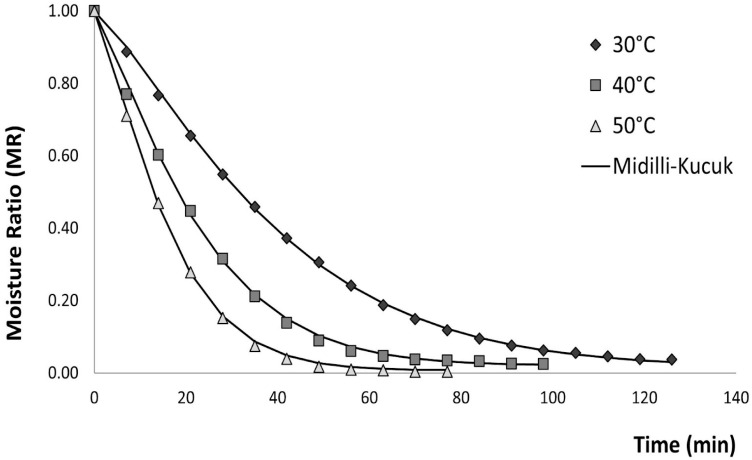
Effect of air-drying temperature (°C) on experimental and predicted drying curves for Midilli–Kucuk model.

Finally, the AICc criterion was applied to identify the most suitable mathematical model. Across all drying temperatures, AICc values ranged from −130.11 to −6.741, with the Midilli–Kucuk model exhibiting the lowest value among the empirical kinetic models. Considering all statistical indicators, this model provided the best fit for describing the convective drying behavior of brown seaweed under the tested temperatures. The Midilli–Kucuk model is the one with the best fit, which is represented in Figure 2.

Other authors, such as [91] Kadam et al. (2015), also state that the Midilli–Kucuk model is one of the best-fit models for representing convective air drying of brown seaweed (*Ascophyllum nodosum*), with values of 0.9683, 0.00178, 1.36867, and −0.00006 for a, k, n, and b, respectively. The fit statistics were 0.999, 0.006, and 0.00004 for R^2^, RMSE, and χ^2^, respectively.

**Figure 3:**
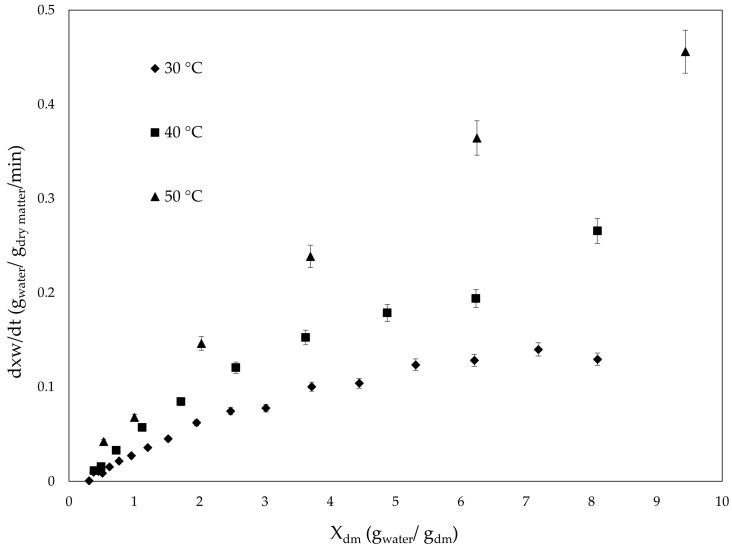
Variation in drying rate (g water/g dry matter/min) versus moisture content (g water/g dry matter) of *Lessonia spicata* undergoing different temperature drying.

Figure 3 shows the relationship between drying rate (DR) and drying time for the temperatures evaluated. In general, it was observed that the velocity decreases as time elapses, suggesting that the hot air oven drying process occurs predominantly in a decreasing velocity phase. This indicates that drying is limited by moisture diffusion within the algal tissue at all temperatures tested [31].

According to [91], drying times have been reported in the range between 210 and 270 min for hot air drying using low air velocity (0.3 m/s at 50 °C) on brown seaweed *A. nodosum*. Similarly, ref. [92], indicated that drying *Alaria esculenta* without continuous air flow and at 40 °C decreased the drying time in a range of 90 to 150 min. Along with this, it is important to recognize that although temperature and other process conditions influence drying time, the structure, size, weight, and state of the algal are important in how heat exchange and water removal from within the matrix occur [28].

### 3.3. Diffusional Parameter and Arrhenius Equation

The traditional method to study mass transfer in a nonstationary state during food drying is by means of the Fick equation (Equation (4)), which allows calculation of the effective diffusivity coefficient of water (D_eff_). In this study, the effective diffusivities of the dried algae at different temperatures were obtained from the slope (k) of the plots of ln (MR) as a function of time in seconds of drying (t) (Equation (5)) for temperatures of 30, 40, and 50 °C. The results of this adjustment allow the calculation of the diffusion coefficients (D_eff_) at each temperature using Equation (6).

The diffusivity coefficient values are 3.1 × 10^−10^, 3.7 × 10^−10^, and 4.7 × 10^−10^ m^2^/s as the temperature increases, with R^2^ values of 0.8667, 0.7642, and 0.7300, respectively. Moreover, the activity of the water molecules increased due to the increase in thermal energy resulting from the temperature increase, leading to the observed increase in effective moisture diffusivity and drying rate [93]. These values are within the range of 10^−12^ to 10^−6^ m^2^/s reported in the literature for food drying [94].

They were similar to the values of 1.53 × 10^−10^ to 5.41 × 10^−10^ m^2^/s reported for hot air drying of *Scenedesmus obliquus* microalgae at 50 °C and 60 °C [95]. This slight difference could be due to the higher initial water content in *L. spicata*, which favors higher diffusion coefficients, since diffusion is facilitated in materials with a higher proportion of water and fewer solids [52]. An increase in the effective diffusivity of water is favored by increasing temperature, which coincides with the final humidity values for the samples at 30, 40, and 50 °C, where a statistical difference between the different values is shown (*p* < 0.05) [96]. Furthermore, studies such as those carried out by [97]. demonstrate this proportional relationship between the increase in temperature and the increase in the value of the diffusion coefficient, where for an increase in temperature from 35 to 50 °C, there was an increase in the values of D_eff_ from 12 to 18 × 10^−12^ m^2^/s in the convective drying of brown algae *Bifurcaria bifurcata.* Another example is the study carried out by [98], where convective drying of two types of seaweed, *Ulva olmoi* and *Oedogonium intermedium*, was carried out, achieving an increase in D_eff_ from 1.2 × 10^−7^ to 1.4 × 10^−7^ m^2^/s for *U. olmoi* and 5.7 × 10^−8^ to 6.7 × 10^−8^ m^2^/s for *O. intermedium* by increasing the temperature from 40 to 50 °C. The diffusivity values obtained were used to adjust Equation (6), allowing estimation of the diffusion coefficient at infinite temperature, thus calculating the parameter D_o_ and the activation energy “E_a_” for moisture diffusion. The results show a good fit with an R^2^ value of 0.911. E_a_ was 11.01 kJ/mol, indicating the amount of energy required to initiate the convective drying process in this macroalgae, *L. spicata*. This value is lower than the range reported for agricultural products, from 18 to 70.2 kJ/mol [96], and similar to the value reported for convective drying of microalgae [99], which implies that drying of Huiro negro requires less energy for water molecules to diffuse to the surface and evaporate.

The value of the activation energy “E_a_” obtained suggests that the sample does not require a high activation energy for the water contained in the algae to begin to diffuse to the surface and evaporate. This could be related to the structural characteristics of the matrix of the material. In addition, in terms of drying speed, a greater thickness of the sample implies a greater distance that the moisture must travel to reach the surface, which translates into slower drying [52].

### 3.4. Energy Requirements and Dynamic Mass Transfer Coefficient (K_G_)

Table 4 presents the energy balance for the Huiro negro dehydration process using forced convective airflow at varying air temperatures, aiming for a final moisture content of 0.3 kg water/kg dry matter. Since the sample was initially subjected to blanching, studies such as the one conducted by [54] indicate that this treatment does not generate a significant reduction in the specific energy required for drying.

Based on electric current consumption measurements, the energy required to heat the air from ambient to operational temperature ranged from 2.28 to 3.05 kWh, with the highest energy demand observed at 30 °C. This was attributed to the longer dehydration time required to achieve the desired moisture content within food [100]. The total energy absorbed by the air during heating ranged from 1.72 to 2.23 kWh, resulting in a heating step efficiency of 56.44 to 97.86%, with the highest efficiency observed at the highest operational temperature. This improved efficiency at elevated temperatures was associated with reduced heat loss to the surroundings and dehydration time when operating at a constant airflow velocity of 2 m/s. During dehydration, water evaporation from the solid food matrix requires energy for phase transition from liquid to vapor.

Our calculations revealed an inverse relationship between operating temperature and the energy required for water evaporation, which ranged from 0.6 to 1.49 kWh. This energy was absorbed from the heated air as it passed over the food surface.

The energy efficiency for water removal varied between 26.78 and 86.35%, with the highest efficiency observed at 30 °C (lowest drying temperature), where 1.49 kWh of energy was used for water removal compared to 1.72 kWh absorbed by the air during heating. A distinctive trend was observed in total energy loss to the environment, with the highest losses occurring at 40 °C, followed by 50 °C, and the lowest at 30 °C.

The specific energy consumption (SEC) for Huiro negro dehydration at a fixed airflow velocity of 2 m/s ranged from 18.29 to 25.25 kWh/kg water, increasing as the operating temperature decreased. Literature reports on microwave, infrared, and hot-air drying show a similar trend: increasing microwave power, air temperature, and IR radiation enhances the thermal gradient and accelerates moisture evaporation, reducing SEC values.

Additionally, while higher air temperatures increase the energy demand for heaters, they also shorten the drying time, ultimately lowering SEC [101]. This suggests that the reduction in drying time has a more significant impact on SEC than the increase in temperature. A clear relationship between total energy costs and overall energy efficiency was observed.

The overall energy efficiency of the Huiro negro dehydration process ranged from 26.21 to 48.73%, decreasing as processing temperatures increased. Previous studies [102] report food dryer energy efficiencies between 30% and 60%, indicating that the actual energy use is approximately 1.5 to 3.0 times the theoretical load. Although the values obtained in this study align with literature, further improvements are needed to enhance cost-effectiveness in food processing. The lowest total energy cost was recorded at 30 °C (5.37 USD/kg), increasing with higher operating temperatures.

The dynamic mass transfer coefficient (K_G_) was estimated during *L. spicata* dehydration under a parallel flow heated air configuration. K_G_ values exhibited a clear dependence on food moisture content, showing dynamic behavior.

Specifically, K_G_ values decreased as moisture content declined, with magnitudes ranging from 10^−7^ to 10^−5^ (kg/m^2^/s/ Pa). A similar behavior has been reported by [58], where K_G_ values decreased as wood moisture content declined, regardless of dimensional geometry or measurement time intervals. Additionally, K_G_ values tended to stabilize when moisture content exceeded 6 (kg water/kg dry matter). Under these conditions, K_G_ exhibited a strong dependence on operational temperature, with higher values recorded at increased temperatures under constant airflow velocity [103]. The average K_G_ values at 30, 40, and 50 °C were 5.29 × 10^−6^, 5.90 × 10^−6^, and 8.71 × 10^−6^ (kg/m/s/Pa), respectively.

### 3.5. Biofunctional Characteristics

#### 3.5.1. Total Phenolic Content

Table 5 presents the effect of convective drying on bioactive compounds and antioxidant capacity in the extract of *L. spicata* brown seaweed. In general, the total phenolic compound (TPC) content decreased as the drying temperature increased, reaching its maximum concentration in the fresh state (1245.87 ± 24.21). However, there were no significant differences between the dehydrated samples (30–50 °C) (*p* > 0.05). The drying process plays a crucial role in determining the total polyphenol content (PFT) of the samples. Moderate drying temperatures can stimulate the plant’s natural stress response, promoting the synthesis of phenolic compounds as part of its tissue repair mechanisms [104]. However, prolonged drying times at lower temperatures (30–40 °C) may lead to a reduction in the available phenolic content compared to fresh samples, as extended exposure to oxidative conditions can degrade these compounds. Furthermore, such low temperatures are often insufficient to fully inactivate oxidative enzymes, allowing partial oxidation of phenolics and consequently resulting in lower PFT values.

The reduction in TPC may also be attributed to the interaction of polyphenols with proteins or to structural changes in the polyphenols that affect their extraction or detection with current methods [105]. It is important to note that brown algae tend to contain higher amounts of polyphenols compared to red and green algae. In addition, it has been observed that TPC is higher in old leaves compared to new leaves, which is consistent with studies such as that of [106] who reported a high correlation between tissue age and TPC for *L. hyperborea*. TPC levels were also reported in fucoid algal species, which could be due to the adaptive response of these species to variable environmental conditions, such as sunlight and climate [107]. The solvent used in the extraction also affects the measured TPC.

Phenolic compounds are generally more soluble in polar organic solvents than in water, and aqueous mixtures of methanol, ethanol, and acetone are recommended for more efficient extraction [108]. For example, in 70% acetone extracts, the TPC values of *A. nodosum*, *F. serratus*, and *F. vesiculosus* were 15.9, 24.0, and 24.2 g PGE/100 g extract, respectively, while aqueous extracts showed slightly lower values [109]. The polarity of any solvent plays a significant role in the extraction of phenolic compounds from plants or fruits. As it can inhibit polyphenoloxidase activity, methanol is usually the most effective solvent for the extraction of polyphenolic compounds [110]. On the other hand, the Folin–Ciocalteu method, widely used for its ability to measure phenolic compounds through redox reactions, has an important limitation: its sensitivity to interferences from other non-phenolic reducing compounds, such as pigments, sugars, proteins, organic acids, and metal chelators. These interferences can generate results that show higher concentrations of phenols in non-polar solvents, where these substances are usually more prevalent [111].

#### 3.5.2. Determination of Carotenoids and Fucoxanthin

The analysis of carotenoid total (CT) and fucoxanthin content (FC) in *L. spicata* macroalgae was carried out on fresh and dehydrated samples at temperatures of 30, 40, and 50 °C. The results obtained indicate that the dehydration process significantly affects the content of these compounds, with a general increase in the concentration of carotenoids and fucoxanthin in the dehydrated samples. Carotenoids are responsible for giving red, yellow, and orange pigmentation to algae and have subgroups such as carotenes and xanthophylls, including fucoxanthin [7].

During convective drying, a significant decrease in carotenoids was observed in dehydrated samples at 30 and 40 °C compared to fresh samples (*p* < 0.05). However, better preservation was recorded at 50 °C, suggesting that higher temperatures favor carotenoid concentration by reducing moisture content, although they may cause decomposition in thermal excess [112]. The data presented in Table 5 show that the fucoxanthin content increased significantly after dehydration. The fresh sample obtained a value of 0.55 µg/100 mL dry basis, while at 50 °C, it reached 2.24 µg/100 mL, increasing by up to three times, approximately. Some factors that affect the concentration of these compounds are the availability in the environment where these macroalgae are found, light and pH, and time of year, among others. All these factors influence the photosynthetic potential of fucoxanthin [113].

The contents of total carotenoids and fucoxanthin are highly correlated, indicating that fucoxanthin represents an important carotenoid in brown algae, as described by [62]. In the macroalga *Lessonia spicata*, fucoxanthin represented 3.7%, 4.3%, and 2.9% in the extracts at these three temperatures studied: 30, 40, and 50 °C, respectively.

However, the fucoxanthin content is lower compared to the total carotenoid value, suggesting the presence of other types of carotenoids in the sample that were not quantified in this study. In addition, these compounds could also contribute to the antioxidant profile of seaweed. Solvent selection is critical for the extraction of carotenoids and fucoxanthin because polar solvents such as methanol have shown high yields for lipophilic compounds [114]. In this study, the extraction of total carotenoids was lower with methanol than reported with DMSO-water, corresponding to values of 28.61 ± 0.79 µg/g dry basis, but consistent with their lipophilic affinity. For its part, fucoxanthin extraction was performed with acetone-water, allowing it to isolate this compound efficiently due to its polar functional groups [115]. This is consistent with the research reported by [114], which shows that methanol was able to extract fucoxanthin from *Padina australis* more optimally than ethanol, DMSO, acetonitrile, and acetone.

On the other hand, the previous blanching performed on *L. spicata* macroalgae samples could have influenced the fucoxanthin content because, according to [116], this compound is released and reacts with proteins, increasing its concentration in the extract. This process could denature proteins in cell walls, increasing permeability and facilitating extraction [117], as in the case of fucoxanthin-chlorophyll a/c binding proteins [118]. Similarly, this treatment inactivates endogenous oxidative enzymes, such as polyphenoloxidase and peroxidase, which decreases the concentration of fucoxanthin, preventing its degradation [119]. However, as reported by [28], it could be preserved by using supplements such as ascorbic acid.

#### 3.5.3. Total Flavonoids Content (TFC)

Flavonoids are natural phenolic compounds with structural characteristics that give them specific biofunctional properties, such as the ability to scavenge free radicals and antioxidant properties. These compounds play a crucial role in the antioxidant defense system of macroalgae, contributing to their bioactivity and potential applications in the nutraceutical and pharmaceutical industries [120]. Table 5 shows the variation in total flavonoid content (TFC) for the temperatures studied. The TFC in fresh seaweed was 0.04 ± 0.004 QE/100 g dry matter. The TPC increased significantly up to the temperature of 40 °C, but higher than this temperature, it decreased. The combination of high drying temperatures and prolonged drying times could destroy some of the phenolic compounds.

In addition, all plant cellular components adhere to each other in the absence of water, possibly making solvent extraction more difficult, i.e., as a result, overall yields would be lower than expected [108]. Thermal degradation of flavonoids is mainly attributed to the cleavage of glycosidic bonds, oxidation reactions, and polymerization processes that occur under prolonged exposure to heat [121]. In addition, plant cellular components tend to aggregate as moisture is removed, which can hinder solvent extraction and reduce flavonoid yields. This phenomenon is particularly pronounced in macroalgae, where polysaccharides such as alginate and fucoidan can encapsulate polyphenols, limiting their availability for extraction [122].

The results obtained in this study indicate that drying at 40 °C is the most favorable condition for preserving flavonoids in *Lessonia spicata*, which is in line with previous studies on other brown algae species. Moderate drying temperatures (40–50 °C) have been reported to be optimal for maintaining the integrity of flavonoid compounds in red macroalgae [123]. In contrast, drying at higher temperatures (>50 °C) may accelerate the decomposition of heat-sensitive flavonoids, leading to a significant reduction in antioxidant capacity.

#### 3.5.4. Characterization of Antioxidant Capacity

The antioxidant capacity (AC) of Huiro negro (*Lessonia spicata*) in fresh and dried states was determined by the DPPH free radical scavenging assay, and the values obtained in Table 5 were expressed as % inhibition, indicating the efficacy of the antioxidant present in the sample to neutralize DPPH free radicals.

Drying resulted in a significant decrease in antioxidant activity, evidenced by the reduction in DPPH free radical scavenging activity (*p* < 0.05). The highest value was observed in the fresh sample, 77% inhibition, which is common in fresh foods, as they retain more antioxidant compounds, which tend to degrade during processing. In comparison, the samples dried at 30 and 40 °C presented lower inhibition percentages, with values of 64 and 59%, respectively.

It is important to note that brown algae tend to contain higher amounts of polyphenols compared to red and green algae. In addition, it has been observed that TPC is higher in old leaves compared to new leaves, which is consistent with studies such as that of [106], who reported a high correlation between tissue age and TPC for *L. hyperborea*. Elevated TPC levels were also reported in fucoid algal species, which could be due to the adaptive response of these species to variable environmental conditions, such as sunlight and climate [107].

The positive correlation between total phenolic content (TPC) and antioxidant capacity observed in *Lessonia spicata* is consistent with findings in other macroalgae, where polyphenols play a dominant role in free radical scavenging [17,124]. The drying process generally results in a decrease in the natural antioxidants present in plant materials, and intense or prolonged heat treatments can cause a significant loss of these compounds, as most of these compounds are relatively unstable [104]. The loss of natural antioxidants could be attributed to different main reasons: changes in chemical structure, the breaking of bonds between compounds and cell walls, partial degradation of cell wall polymers due to oxidative changes, and thermal degradation of the compounds themselves caused by inactivated oxidative enzymes [104]. However, in the sample dried at 50 °C, the inhibition percentage increased to 74%, a value close to that of the fresh sample. This suggests that specific antioxidant compounds are present that are less sensitive to high temperatures or to the formation of new antioxidant compounds during the drying process.

#### 3.5.5. Cytotoxic Effect on Cell Viability

**Figure 4:**
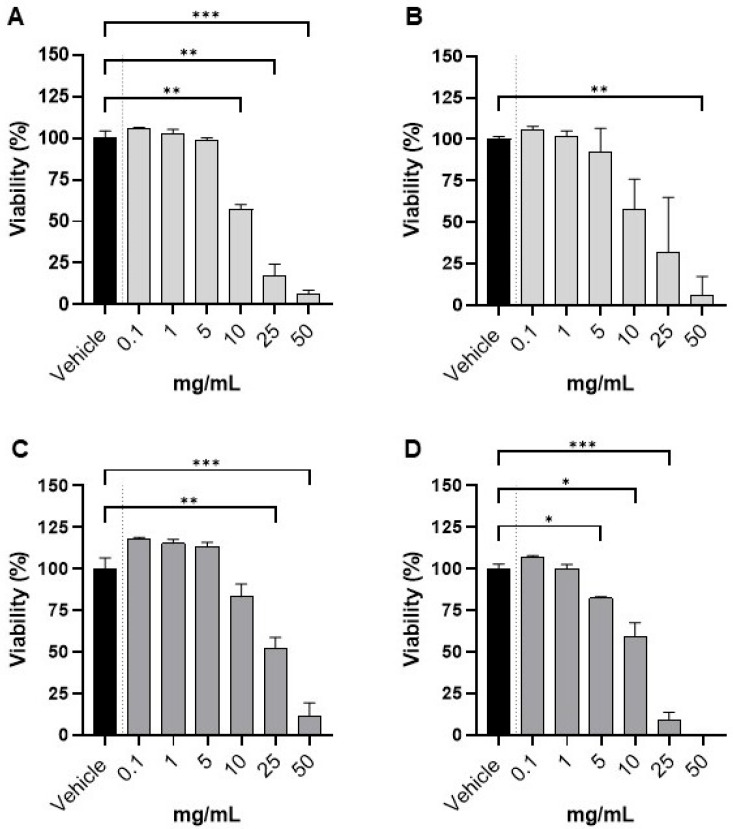
Cell viability in GES 1 and AGS after exposure to Lessonia spicata extracts: dose–response curves. Cell viability (%) of GES-1 (panels (**A**,**B**)) and AGS (panels (**C**,**D**)) after 24 h exposure to Lessonia spicata extracts at 0.1–50 mg/mL, where panels (**A**,**C**) show fresh extract and panels (**B**,**D**) show extract from biomass dried at 50 °C. Vehicle control was set as 100% viability. At 50 mg/mL, the 50 °C extract reduced viability to ~0% (panel (**D**)), precluding statistical analysis at this point. (* *p* < 0.05; ** *p* < 0.01, and *** *p* < 0.001).

Subsequently, we evaluated cell viability by trypan blue exclusion after 24 h exposure to fresh and 50 °C extracts of *Lessonia spicata* in the normal gastric epithelial cell line GES-1 and the gastric tumor-derived AGS cell line (Figure 4). Both types of extracts exhibited minimal cytotoxicity at concentrations up to 1 mg/mL (≥90% viability compared to the 60% methanol vehicle).

In GES-1 cells, viability remained comparable to vehicle for both the fresh (panel A) and 50 °C-dried (panel B) extracts at low concentrations (0.1–1 mg/mL), with no statistically significant differences. Only at higher concentrations (≥10 mg/mL) did we observe a significant, dose-dependent reduction in viability, with the greatest effect observed at 50 mg/mL (*p* < 0.05–0.01 vs. vehicle). In AGS tumor-derived cells, the response was similar but revealed greater sensitivity, particularly for the extract derived from biomass dried at 50 °C (panel D). Here, significant reductions in viability were apparent from 5 mg/mL onward and became more pronounced at concentrations between 10 and 50 mg/mL. The fresh extract (panel C) also induced a decrease in viability, though significant effects were observed only at the highest doses tested. Of note, exposure to the 50 °C extract at 50 mg/mL reduced AGS cell viability to nearly 0%, which precluded post hoc statistical analysis at this concentration.

These results, together with the higher fucoxanthin content in the 50 °C extract, support the conclusion that Lessonia spicata extracts possess low cytotoxicity and high biocompatibility at physiologically relevant concentrations. Importantly, the extracts selectively impaired viability in tumor-derived AGS cells at high doses, while normal gastric GES-1 cells were largely unaffected except at supraphysiological levels. Collectively, these findings underline the safety profile of these seaweed extracts and suggest their suitability as food ingredients, as they preserve normal cell viability at low to moderate concentrations while conferring greater cytotoxicity toward gastric tumor cells only at much higher doses.

## 4. Conclusions

The present study demonstrated that among the empirical models evaluated to predict the drying kinetics of Huiro negro (*Lessonia spicata*), the Midilli–Kucuk model offered the highest accuracy and statistical reliability. From an energy standpoint, the highest OTE value along with the lowest total energy cost was achieved at 30 °C. However, drying at 50 °C represented the most favorable compromise between product quality and functionality, preserving antioxidant capacity, concentrating macronutrients, and maintaining elevated fucoxanthin levels. Extract obtained under this condition exhibited low cytotoxicity toward GES-1 and AGS gastric cells at concentrations ≤ 1 mg/mL, maintaining cell viability above 90%, although higher concentrations reduced viability in a dose-dependent manner. Further improvements in drying technology are required to enhance both energy efficiency and the chemical stability of bioactive compounds. Emerging food processing technologies—such as vacuum, infrared, or microwave-assisted drying—could improve thermal efficiency and preservation of bioactive compounds, enabling superior preservation of L. spicata towards a sustainable, functional seaweed-based product for the food and nutraceutical industries.

## Figures and Tables

**Table 1:** Semi-empirical mathematical models used to describe thin-layer drying kinetics of brown seaweed (*Lessonia spicata*) undergoing hot-air forced convection dehydration.

Model Name	Mathematical Expression	Reference
Page	MR = exp (−k t^n^)	[45]
Weibull	MR = exp [−(t/β) ^α^]	[46]
Wang and Singh	MR = a t^2^ + b t + 1	[47]
Henderson and Pabis	MR = n exp (−k t)	[47]
Midilli–Kucuk	MR = a exp (−k t^n^) + b t	[48]
Logarithmic	MR = a exp (−k t) + c	[49]
Verma	MR = a exp (−k t) + (1−a) exp (−c t)	[50]
Verma modified	MR = a exp (−b t^n^) + (1−a) exp (−c t^n^)	[50]

MR is the moisture ratio, t is the drying time, and the letters a, b, c, k, n, α and β are model parameters.

**Table 2:** Proximate composition analysis of fresh and dehydrated samples.

Parameters (g/100 g Wet Matter)	Fresh *	Temperature (°C)		
		30 °C	40 °C	50 °C
Moisture	87.5 ± 0.03 ^a^	14.28 ± 0.13 ^b^	11.92 ± 0.07 ^c^	9.1 ± 0.02 ^d^
Lipid	0.19 ± 0.09 ^a^	1.38 ± 0.05 ^c^	1.7 ± 0.06 ^b^	1.31 ± 0.01 ^c^
Protein	1.72 ± 0.25 ^b^	13.21 ± 0.01 ^b^	13.16 ± 0.06 ^b^	14.48 ± 0.67 ^a^
Ash	1.81 ± 0.01 ^a^	11.16 ± 0.02 ^c^	11.16 ± 0.02 ^c^	12.94 ± 0.01 ^b^
Total dietary fiber	8.71 ± 3.65 ^a^	59.8 ± 0.16 ^b^	61.32 ± 0.90 ^b^	62.35 ± 0.48 ^b^
Available carbohydrates	0.23 ± 0.01 ^c^	0.18 ± 0.10 ^c^	1.5 ± 0.17 ^a^	1.02 ± 0.24 ^b^

Results are expressed as mean ± standard deviation and were analyzed in triplicate. (*n* = 3). Different letters among columns (a–d) for each row indicate statistically significant differences (*p* < 0.05). * Values expressed in g/100 g wet basis.

**Table 3:** Statistical parameters for convective drying of brown seaweed (Lessonia spicata) at 30, 40, and 50 °C.

Temperature	Models	Parameters	R^2^	SSE	RMSE	χ^2^	AICc
30 °C	Page	n = 1.350, k = 0.007	0.998	0.998	0.008	1.053	−62.387
	Weibull	α = 1.350, β = 37.696	0.998	0.998	0.008	1.053	−62.379
	Wang-Singh	a = 0.0001, b = −0.019	0.998	1.029	0.027	1.086	−54.089
	Henderson and Pabis	n = 1.0572, k = 0.0257	0.995	1.077	0.053	1.137	−80.657
	Midilli–Kucuk	a = 0.993, k = 0.009, n = 1.264, b = 0.0001	0.999	0.001	0.0005	0.00004	−130.119
	Logarithmic	a = 1.106, k = 0.023, c = −0.054	0.999	0.008	0.022	0.0009	−87.091
	Verma	a = 2.002, k = 0.035, c = 0.055	0.999	0.001	0.006	0.00004	−119.262
	Verma modified	a = 4.240, b = 0.039, c = 0.046, n = 1.000	0.999	0.001	0.007	0.00004	−100.971
40 °C	Page	n = 1.276, k = 0.019	0.976	0.992	0.025	1.063	−56.661
	Weibull	α = 1.276, β = 22.149	0.976	0.992	0.025	1.063	−55.063
	Wang-Singh	a = 0.0002, b = −0.0281	0.986	0.726	0.181	1.181	−41.230
	Henderson and Pabis	n = 1.576, k = 0.070	0.938	0.930	0.049	0.996	−6.741
	Midilli–Kucuk	a = 0.989, k = 0.020, n = 1.225, b = 0.0002	0.999	0.002	0.012	0.0002	−81.686
	Logarithmic	a = 1.040, k = 0.041, c = −0.016	0.995	0.007	0.024	0.09160	−64.566
	Verma	a = 2.269, k = 0.059, c = 0.082	0.998	0.002	0.014	0.08352	−80.896
	Verma modified	a = 3.830, b = 0.063, c = 0.075, n = 0.998	0.998	0.002	0.014	0.09112	−76.935
50 °C	Page	n = 1.328, k: 0.024	0.975	0.993	0.020	1.083	−66.495
	Weibull	α = 1.328, β: 16.588	0.975	0.993	0.020	1.083	−66.554
	Wang-Singh	a = 0.0003, b: −0.0379	0.978	0.082	0.082	0.949	−11.118
	Henderson and Pabis	n = 1.0352, k: 0.0732	0.975	0.968	0.031	1.056	−46.066
	Midilli–Kucuk	a = 0.996, k = 0.027, n = 1.270, b = 0.00001	0.999	0.001	0.007	0.00010	−78.478
	Logarithmic	a = 1.058, k = 0.058, c = −0.032	0.995	0.006	0.026	0.11190	−47.598
	Verma	a = 6.467, k = 0.102, c = 0.115	0.999	0.001	0.008	0.12506	−77.456
	Verma modified	a = 4.378, b = 0.034, c = 0.031, n = 1.083	0.998	0.002	0.016	0.12525	−55.915

Coefficient of determination (R^2^); sum of square error (SSE), Root mean square error (RMSE), Chi-square (χ^2^), and Akaike Information Criterion with bias correction (AICc).

**Table 4:** Energy balance for Huiro negro dehydration using forced convective airflow (2 m/s) in a parallel air-food configuration for X final dry matter = 0.3 kg water/kg dry matter **.

	Energy Supplied by Heating	Energy Absorbed by Air	Energy Lost in Air Heating	Energy Efficiency Air Heating	Energy for Water Evaporation	Energy for Water Remotion	Energy Lost to Environment	SEC	Total Cost of Energy *	OTE
T (°C)	Q_available_ (kWh)	Q_air_ (kWh)	Q_lost, a_._h_ (kWh)	(%)	Q_water_ (kWh)	(%)	Q_total lost_ (kWh)	Q_specific_ (kWh/kg)	USD/kg	(%)
30	3.05	1.72	1.33	56.44	1.49	86.35	1.57	25.24	5.37	48.73
40	2.79	2.12	0.67	75.95	0.84	39.74	1.95	22.60	6.47	30.19
50	2.28	2.23	0.05	97.86	0.60	26.78	1.69	18.29	7.20	26.21

* The cost of energy was calculated to be 0.4 USD/kWh. Source: SASIPA Spa. ** All values were standardized when achieving a similar moisture content value during dehydration.

**Table 5:** Effect of different drying temperatures on functional properties of Huiro negro.

		Temperatures (°C)		
Analysis	Fresh	30	40	50
TPC (mg EGA/100 g d.m.)	1245.87 ± 24.21 ^a^	702.58 ± 8.13 ^b^	801.73 ± 4.51 ^b^	729.81 ± 4.76 ^b^
TFC (mg EQ/100 g d.m.)	37.60 ± 3.59 ^c^	83.14 ± 0.88 ^b^	109.24 ± 8.32 ^a^	92.13 ± 7.23 ^b^
CT (µg/100 mL d.m.)	561.82 ± 43.71 ^a^	45.75 ± 0.03 ^b^	35.33 ± 0.31 ^b^	77.58 ± 4.04 ^b^
FC (µg/100 mL d.m.)	0.55 ± 0.02 ^d^	1.69 ± 0.02 ^b^	1.53 ± 0.01 ^c^	2.24 ± 0.05 ^a^
AC (% of inhibition)	76.93 ± 0.50 ^a^	63.80 ± 4.10 ^b^	59.11 ± 1.22 ^b^	73.73 ± 0.86 ^a^

TPC: Total phenolic content, TFC: Total flavonoid content, CT: Carotenoid content, FC: Fucoxanthin, AC: Antioxidant capacity by the DPPH method. The letters (a–d) for each column indicate significant differences for each row (*p* < 0.05). Results are expressed as mean ± standard deviation and were analyzed in triplicate (n = 3).

## Data Availability

The original contributions presented in this study are included in the article. Further inquiries can be directed to the corresponding author.

## References

[1] Malafronte L., Yilmaz-Turan S., Krona A., Martinez-Sanz M., Vilaplana F., Lopez-Sanchez P. (2021). Macroalgae Suspensions Prepared by Physical Treatments: Effect of Polysaccharide Composition and Microstructure on the Rheological Properties. Food Hydrocoll..

[2] Hughes M.H., Fernández Severini M., Scodelaro Bilbao P.G. (2024). Editorial: World’s Oceans: Opportunities and Challenges Looking under the Sea. Front. Mar. Sci..

[3] Polat S., Trif M., Rusu A., Šimat V., Čagalj M., Alak G., Meral R., Özogul Y., Polat A., Özogul F. (2023). Recent Advances in Industrial Applications of Seaweeds. Crit. Rev. Food Sci. Nutr..

[4] Koyande A.K., Chew K.W., Manickam S., Chang J.S., Show P.L. (2021). Emerging Algal Nanotechnology for High-Value Compounds: A Direction to Future Food Production. Trends Food Sci. Technol..

[5] Véliz K., Toledo P., Araya M., Gómez M.F., Villalobos V., Tala F. (2022). Chemical Composition and Heavy Metal Content of Chilean Seaweeds: Potential Applications of Seaweed Meal as Food and Feed Ingredients. Food Chem..

[6] Chekanov K., Litvinov D., Fedorenko T., Chivkunova O., Lobakova E. (2021). Combined Production of Astaxanthin and β-Carotene in a New Strain of the Microalga *Bracteacoccus aggregatus* BM5/15 (IPPAS C-2045) Cultivated in Photobioreactor. Biology.

[7] Pereira L. (2021). Macroalgae. Encyclopedia.

[8] Rapoport A., Guzhova I., Bernetti L., Buzzini P., Kieliszek M., Kot A.M. (2021). Carotenoids and Some Other Pigments from Fungi and Yeasts. Metabolites.

[9] Prabhu M.S., Israel A., Palatnik R.R., Zilberman D., Golberg A. (2020). Integrated Biorefinery Process for Sustainable Fractionation of Ulva Ohnoi (Chlorophyta): Process Optimization and Revenue Analysis. J. Appl. Phycol..

[10] Nardelli A.E., Visch W., Wright J.T., Hurd C.L. (2023). Concise Review of Genus *Lessonia bory*. J. Appl. Phycol..

[11] Buschmann A.H., Carmen Hernández-González M., Aroca G., Gutierrez A. Seaweed Farming in Chile: A Review. https://www.globalseafood.org/advocate/seaweed-farming-in-chile-a-review/.

[12] Tagliapietra B.L., Clerici M.T.P.S. (2023). Brown Algae and Their Multiple Applications as Functional Ingredient in Food Production. Food Res. Int..

[13] Ghallab D.S., Ibrahim R.S., Mohyeldin M.M., Shawky E. (2024). Marine Algae: A Treasure Trove of Bioactive Anti-Inflammatory Compounds. Mar. Pollut. Bull..

[14] Rajendra Prasad A., Shankar R., Patil C.K., Karthick A., Kumar A., Rahim R. (2021). Performance Enhancement of Solar Photovoltaic System for Roof Top Garden. Environ. Sci. Pollut. Res..

[15] Priya A., Anusha G., Thanigaivel S., Karthick A., Mohanavel V., Velmurugan P., Balasubramanian B., Ravichandran M., Kamyab H., Kirpichnikova I.M. (2023). Removing Microplastics from Wastewater Using Leading-Edge Treatment Technologies: A Solution to Microplastic Pollution—A Review. Bioprocess. Biosyst. Eng..

[16] Remya R.R., Samrot A.V., Kumar S.S., Mohanavel V., Karthick A., Chinnaiyan V.K., Umapathy D., Muhibbullah M. (2022). Bioactive Potential of *Brown algae*. Adsorpt. Sci. Technol..

[17] Aminina N.M., Karaulova E.P., Vishnevskaya T.I., Yakush E.V., Kim Y.K., Nam K.H., Son K.T. (2020). Characteristics of Polyphenolic Content in Brown Algae of the Pacific Coast of Russia. Molecules.

[18] Galasso C., Gentile A., Orefice I., Ianora A., Bruno A., Noonan D.M., Sansone C., Albini A., Brunet C. (2019). Microalgal Derivatives as Potential Nutraceutical and Food Supplements for Human Health: A Focus on Cancer Prevention and Interception. Nutrients.

[19] Djaeni M., Utari F.D., Sasongko S.B., Kumoro A.C. (2018). Evaluation of Food Drying with Air Dehumidification System: A Short Review. IOP Conf. Ser. Earth Environ. Sci..

[20] Santos S.d.J.L., Canto H.K.F., da Silva L.H.M., Rodrigues A.M.d.C. (2022). Characterization and Properties of *Purple yam* (Dioscorea Trifida) Powder Obtained by Refractance Window Drying. Dry. Technol..

[21] Mayol A.P., Cruz A.L., Calapatia A., Pancho J.A., Peckson N., Sanchez L., Villoria P., Culaba A. Investigation of the Drying Characteristics of Seaweed Using Offshore Dryer. Proceedings of the IEEE 11th International Conference on Humanoid, Nanotechnology, Information Technology, Communication and Control, Environment, and Management (HNICEM).

[22] Charles A.L., Sridhar K., Alamsjah M.A. (2020). Effect of Drying Techniques on Color and Bioactive Potential of Two Commercial Edible Indonesian Seaweed Cultivars. J. Appl. Phycol..

[23] Mello R.E., Fontana A., Mulet A., Corrêa J.L.G., Cárcel J.A. (2021). PEF as Pretreatment to Ultrasound-Assisted Convective Drying: Influence on Quality Parameters of Orange Peel. Innov. Food Sci. Emerg. Technol..

[24] Prakash O., Kumar A., Prakash O., Kumar A. (2017). Solar Drying Technology: Concept, Design, Testing, Modeling, Economics, and Environment.

[25] Baldán Y., Fernandez A., Urrutia A.R., Fabani M.P., Rodriguez R., Mazza G. (2020). Non-Isothermal Drying of Bio-Wastes: Kinetic Analysis and Determination of Effective Moisture Diffusivity. J. Environ. Manag..

[26] Uribe E., Vega-Gálvez A., Vásquez V., Lemus-Mondaca R., Callejas L., Pastén A. (2017). Hot-Air Drying Characteristics and Energetic Requirement of the Edible *Brown seaweed* Durvillaea Antarctica. J. Food Process Preserv..

[27] EL-Mesery H.S. (2022). Improving the Thermal Efficiency and Energy Consumption of Convective Dryer Using Various Energy Sources for Tomato Drying. Alex. Eng. J..

[28] Santhoshkumar P., Yoha K.S., Moses J.A. (2023). Drying of Seaweed: Approaches, Challenges and Research Needs. Trends Food Sci. Technol..

[29] Kamble M.G., Singh A., Kumar N., Dhenge R.V., Rinaldi M., Chinchkar A.V. (2022). Semi-Empirical Mathematical Modeling, Energy and Exergy Analysis, and Textural Characteristics of Convectively Dried *Plantain banana* Slices. Foods.

[30] Le Loeuff J., Boy V., Morançais P., Hardouin K., Bourgougnon N., Lanoisellé J.L. (2023). Air Drying of Brown Algae *Sargassum*: Modelling and Recovery of Valuable Compounds. J. Appl. Phycol..

[31] Gupta S., Cox S., Abu-Ghannam N. (2011). Effect of Different Drying Temperatures on the Moisture and Phytochemical Constituents of Edible Irish Brown Seaweed. LWT.

[32] Abbaspour-Gilandeh Y., Jahanbakhshi A., Kaveh M. (2020). Prediction Kinetic, Energy and Exergy of Quince under Hot Air Dryer Using ANNs and ANFIS. Food Sci. Nutr..

[33] Hernández A., González-Moya M., Márquez A., Acevedo L. (2024). Review Microalgae Drying: A Comprehensive Exploration from Conventional Air Drying to Microwave Drying Methods. Future Foods.

[34] El-Mesery H.S., Farag H.A., Kamel R.M., Alshaer W.G. (2023). Convective Hot Air Drying of Grapes: Drying Kinetics, Mathematical Modeling, Energy, Thermal Analysis. J. Therm. Anal. Calorim..

[35] Zhu X., Healy L., Zhang Z., Maguire J., Sun D.W., Tiwari B.K. (2021). Novel Postharvest Processing Strategies for Value-Added Applications of Marine Algae. J. Sci. Food Agric..

[36] Moran A.E., Farrell M., Cazabon D., Sahoo S.K., Mugrditchian D., Pidugu A., Chivardi C., Walbaum M., Alemayehu S., Isaranuwatchai W. (2022). Building the Health-Economic Case for Scaling up the WHO-HEARTS Hypertension Control Package in Low- and Middle-Income Countries. Rev. Panam. De. Salud Publica/Pan Am. J. Public Health.

[37] Xu B., Zhang M., Bhandari B. (2014). Temperature and Quality Characteristics of Infrared Radiation-Dried Kelp at Different Peak Wavelengths. Dry. Technol..

[38] Ijarotimi S.O., Keshinro O.O. (2013). Determination of Nutrient Composition and Protein Quality of Potential Complementary Foods Formulated from the Combination of Fermented Popcorn, *African locust* and *Bambara groundnut* Seed Flour. Pol. J. Food Nutr. Sci..

[39] Dubois M., Gilles K.A., Hamilton J.K., Rebers P.A., Smith F. (1956). Colorimetric Method for Determination of Sugars and Related Substances. Anal. Chem..

[40] Angell A.R., Mata L., de Nys R., Paul N.A. (2016). The Protein Content of Seaweeds: A Universal Nitrogen-to-Protein Conversion Factor of Five. J. Appl. Phycol..

[41] Asp N.-G. (1987). Dietary Fibre-Definition, Chemistry and Analytical Determination. Mol. Asp. Med..

[42] Chirapart A., Praiboon J., Puangsombat P., Pattanapon C., Nunraksa N. (2014). Chemical Composition and Ethanol Production Potential of *Thai seaweed* Species. J. Appl. Phycol..

[43] Vega-Gálvez A., Uribe E., Gómez-Pérez L.S., García V., Mejias N., Pastén A. (2022). Drying Kinetic Modeling and Assessment of Mineral Content, Antimicrobial Activity, and Potential α-Glucosidase Activity Inhibition of a Green Seaweed (*Ulva* spp.) Subjected to Different Drying Methods. ACS Omega.

[44] Ren Q., Fang J., Zhao Y. (2025). Prediction Method of Tangerine Peel Drying Moisture Ratio Based on KAN-BiLSTM and Multimodal Feature Fusion. Appl. Sci..

[45] Simpson R., Ramírez C., Nuñez H., Jaques A., Almonacid S. (2017). Understanding the Success of Page’s Model and Related Empirical Equations in Fitting Experimental Data of Diffusion Phenomena in Food Matrices. Trends Food Sci. Technol..

[46] Issis Q.F., Antonio V.G., Elsa U., Valeria V., Nicole C., Jacqueline P. (2019). Vacuum Drying Application to Maqui (*Aristotelia chilensis* [Mol] Stuntz) Berry: Weibull Distribution for Process Modelling and Quality Parameters. J. Food Sci. Technol..

[47] Inyang U.E., Oboh I.O., Etuk B.R. (2018). Kinetic Models for Drying Techniques—Food Materials. Adv. Chem. Eng. Sci..

[48] Midilli A., Kucuk H. (2003). Mathematical Modeling of Thin Layer Drying of Pistachio by Using Solar Energy. Energy Convers. Manag..

[49] Afolabi T.J., Tunde-Akintunde T.Y., Adeyanju J.A. (2015). Mathematical Modeling of Drying Kinetics of Untreated and Pretreated Cocoyam Slices. J. Food Sci. Technol..

[50] Wang W., Chen J., Jin N., Wang H., Wang L., Wu J. (2024). Thin-Layer Drying Model, Drying Rate, and Effective Water Diffusion Coefficient of Pelleted Feed. Int. J. Chem. Eng..

[51] Ebiyeritei E.W. (2023). Determination of Drying Kinetics of Periwinkle Meat (*Turritella communis*) by Application of Some Thin Layer Models. Agric. Eng. Int. CIGR J..

[52] Akpan G.E., Aregbesola O.A., Olagunju T.M. (2024). Analysis of Drying Kinetics and Energy Consumption of Brine Pretreated Freshwater Prawn Fillets. J. Food Process Eng..

[53] Ndukwu M.C., Dirioha C., Abam F.I., Ihediwa V.E. (2017). Heat and Mass Transfer Parameters in the Drying of Cocoyam Slice. Case Stud. Therm. Eng..

[54] Olagunju T.M., Taiwo K.A. (2020). Modeling and Optimization of the Grilling Process of Beef Suya. J. Food Process Preserv..

[55] Beigi M. (2016). Energy Efficiency and Moisture Diffusivity of Apple Slices during Convective Drying. Food Sci. Technol..

[56] Amedor E.N., Sarpong F., Bordoh P.K., Frimpong Boateng E., Owusu-Kwarteng J. (2024). Modelling Convectional Oven Drying Characteristics and Energy Consumption of Dehydrated Yam (*Dioscorea rotundata*) Chips. Heliyon.

[57] Ananias R.A., Perez P., Salinas C., Elustondo D. (2013). Drying Schedules for Canelo Wood. Dry. Technol..

[58] Lerman P., Scheepers G. (2023). Determination of a Mass-Transfer Coefficient for Wood Drying by Means of Thermography. Wood Mater. Sci. Eng..

[59] Ganesan P., Kumar C.S., Bhaskar N. (2008). Antioxidant Properties of Methanol Extract and Its Solvent Fractions Obtained from Selected Indian *Red seaweeds*. Bioresour. Technol..

[60] Li B.B., Smith B., Hossain M.M. (2006). Extraction of Phenolics from Citrus Peels: I. Solvent Extraction Method. Sep. Purif. Technol..

[61] Rafi M., Febriany S., Wulandari P., Suparto I.H., Ridwan T., Rahayu S., Siswoyo D.M. (2018). Total Phenolics, Flavonoids, and Anthocyanin Contents of *Six vireya rhododendron* from Indonesia and Evaluation of Their Antioxidant Activities. J. Appl. Pharm. Sci..

[62] Osório C., Machado S., Peixoto J., Bessada S., Pimentel F.B., Alves R.C., Oliveira M.B.P.P. (2020). Pigments Content (Chlorophylls, Fucoxanthin and Phycobiliproteins) of Different Commercial Dried Algae. Separations.

[63] Alzagameem A., Khaldi-Hansen B.E., Büchner D., Larkins M., Kamm B., Witzleben S., Schulze M. (2018). Lignocellulosic Biomass as Source for Lignin-Based Environmentally Benign Antioxidants. Molecules.

[64] Sandoval-Bórquez A., Polakovicova I., Carrasco-Véliz N., Lobos-González L., Riquelme I., Carrasco-Avino G., Bizama C., Norero E., Owen G.I., Roa J.C. (2017). MicroRNA-335-5p Is a Potential Suppressor of Metastasis and Invasion in Gastric Cancer. Clin. Epigenetics.

[65] Lin Q., Zong X., Lin H., Huang X., Wang J., Nie S. (2023). Based on Quality, Energy Consumption Selecting Optimal Drying Methods of Mango Slices and Kinetics Modelling. Food Chem. X.

[66] Okonkwo C.E., Moses O.I., Nwonuma C., Abiola T., Benjamin B.O., Folorunsho J.O., Olaniran A.F., Pan Z. (2022). Infrared and Microwave as a Dry Blanching Tool for Irish Potato: Product Quality, Cell Integrity, and Artificial Neural Networks (ANNs) Modeling of Enzyme Inactivation Kinetic. Innov. Food Sci. Emerg. Technol..

[67] López G.G., Brousse M.M., Linares A.R. (2023). Kinetic Modelling of Total Phenolic Compounds from *Ilex* Paraguariensis (St. Hil.) Leaves: Conventional and Ultrasound Assisted Extraction. Food Bioprod. Process..

[68] Serafin J., Dziejarski B. (2023). Application of Isotherms Models and Error Functions in Activated Carbon CO_2_ Sorption Processes. Microporous Mesoporous Mater..

[69] Spiess A.-N., Neumeyer N. (2010). An Evaluation of R2 as an Inadequate Measure for Nonlinear Models in Pharmacological and Biochemical Research: A Monte Carlo Approach. BMC Pharmacol..

[70] Pardilhó S., Cotas J., Pereira L., Oliveira M.B., Dias J.M. (2022). Marine Macroalgae in a Circular Economy Context: A Comprehensive Analysis Focused on Residual Biomass. Biotechnol. Adv..

[71] Zhang Y., Hawboldt K., MacQuarrie S. (2024). Extraction of Bioactive Compounds from Beach-Cast Brown Algae: A Review on Accelerated Solvent Extraction and Subcritical Water Extraction. RSC Sustain..

[72] Saji S., Hebden A., Goswami P., Du C. (2022). A Brief Review on the Development of Alginate Extraction Process and Its Sustainability. Sustainability.

[73] Silva A., Rodrigues C., Garcia-Oliveira P., Lourenço-Lopes C., Silva S.A., Garcia-Perez P., Carvalho A.P., Domingues V.F., Barroso M.F., Delerue-Matos C. (2021). Screening of Bioactive Properties in *Brown algae* from the Northwest Iberian Peninsula. Foods.

[74] Aljabri H., Cherif M., Siddiqui S.A., Bounnit T., Saadaoui I. (2023). Evidence of the Drying Technique’s Impact on the Biomass Quality of *Tetraselmis subcordiformis* (*Chlorophyceae*). Biotechnol. Biofuels Bioprod..

[75] Fontana A.J. (2020). D: Minimum Water Activity Limits for Growth of Microorganisms. Water Activity in Foods: Fundamentals and Applications.

[76] Makkar H.P.S., Tran G., Heuzé V., Giger-Reverdin S., Lessire M., Lebas F., Ankers P. (2016). Seaweeds for Livestock Diets: A Review. Anim. Feed. Sci. Technol..

[77] Dhandwal A., Bashir O., Malik T., Salve R.V., Dash K.K., Amin T., Shams R., Wani A.W., Shah Y.A. (2025). Sustainable Microalgal Biomass as a Potential Functional Food and Its Applications in Food Industry: A Comprehensive Review. Environ. Sci. Pollut. Res..

[78] Peñalver R., Lorenzo J.M., Ros G., Amarowicz R., Pateiro M., Nieto G. (2020). Seaweeds as a Functional Ingredient for a Healthy Diet. Mar. Drugs.

[79] Kasimala M., Tsighe N., Kasimala M.B., Mebrahtu L., Mehari A. (2017). Proximate Composition of Three Abundant Species of Seaweeds from Red Sea Coast in Massawa, Eritrea. J. Algal Biomass Util..

[80] Martínez–Hernández G.B., Castillejo N., Carrión–Monteagudo M.d.M., Artés F., Artés-Hernández F. (2018). Nutritional and Bioactive Compounds of Commercialized Algae Powders Used as Food Supplements. Food Sci. Technol. Int..

[81] Henchion M., Hayes M., Mullen A.M., Fenelon M., Tiwari B. (2017). Future Protein Supply and Demand: Strategies and Factors Influencing a Sustainable Equilibrium. Foods.

[82] Olsson J., Toth G.B., Albers E. (2020). Biochemical Composition of Red, Green and Brown Seaweeds on the Swedish West Coast. J. Appl. Phycol..

[83] Neto R.T., Marçal C., Queirós A.S., Abreu H., Silva A.M.S., Cardoso S.M. (2018). Screening of *Ulva rigida*, *Gracilaria* sp., Fucus Vesiculosus and *Saccharina latissima* as Functional Ingredients. Int. J. Mol. Sci..

[84] Sappati P.K., Nayak B., VanWalsum G.P., Mulrey O.T. (2019). Combined Effects of Seasonal Variation and Drying Methods on the Physicochemical Properties and Antioxidant Activity of Sugar Kelp (*Saccharina latissima*). J. Appl. Phycol..

[85] Elhassaneen Y., ELBassouny G., Emam O., Alsobky F.A. (2024). Brown Algae Is a Natural Source Rich in Nutrients and Bioactive Compounds: Application in Balady Bread. Am. J. Food Sci. Technol..

[86] Nakhate P., van der Meer Y. (2021). A Systematic Review on Seaweed Functionality: A Sustainable Bio-Based Material. Sustainability.

[87] Akter A., Sobuj M.K.A., Islam M.S., Chakroborty K., Tasnim N., Ayon M.H., Hossain M.F., Rafiquzzaman S.M. (2024). Seaweed Polysaccharides: Sources, Structure and Biomedical Applications with Special Emphasis on Antiviral Potentials. Future Foods.

[88] Olanipekun B.F., Tunde-Akintunde T.Y., Oyelade O.J., Adebisi M.G., Adenaya T.A. (2015). Mathematical Modeling of Thin-Layer Pineapple Drying. J. Food Process Preserv..

[89] Le Loeuff J., Boy V., Morançais P., Colinart T., Bourgougnon N., Lanoisellé J.L. (2021). Mathematical Modeling of Air Impingement Drying of the Brown Algae *Sargassum muticum* (*Fucales*). Chem. Eng. Technol..

[90] Lemus-Mondaca R., Vega-Gálvez A., Moraga N.O., Astudillo S. (2015). Dehydration of *Stevia rebaudiana* Bertoni Leaves: Kinetics, Modeling and Energy Features. J. Food Process Preserv..

[91] Kadam S.U., Tiwari B.K., O’Donnell C.P. (2015). Effect of Ultrasound Pre-Treatment on the Drying Kinetics of Brown Seaweed *Ascophyllum nodosum*. Ultrason. Sonochem..

[92] Sørensen J.S., Rugh van Reeuwijk S., Bartle R.S., Hansen L.T. (2023). Inactivation of *Salmonella typhimurium* During Low Heat Convection Drying of Winged Kelp (Alaria Esculenta). LWT.

[93] Xiao H.W., Pang C.L., Wang L.H., Bai J.W., Yang W.X., Gao Z.J. (2010). Drying Kinetics and Quality of Monukka Seedless Grapes Dried in an Air-Impingement Jet Dryer. Biosyst. Eng..

[94] Erbay Z., Icier F. (2010). A Review of Thin Layer Drying of Foods: Theory, Modeling, and Experimental Results. Crit. Rev. Food Sci. Nutr..

[95] Simioni T., Quadri M.B., Derner R.B. (2019). Drying of *Scenedesmus obliquus*: Experimental and Modeling Study. Algal Res..

[96] Doymaz I., Özdemir Ö. (2014). Effect of Air Temperature, Slice Thickness and Pretreatment on Drying and Rehydration of Tomato. Int. J. Food Sci. Technol..

[97] Arufe-Vilas S., Sineiro J., Chenlo F., Moreira R. Convective Air Drying of Brown Seaweed *Bifurcaria bifurcata* in Thin Layer Configuration. Proceedings of the 21st International Drying Symposium.

[98] Walker C., Cole A., Antunes E., Sheehan M. (2020). Equilibrium Moisture and Drying Kinetics Modelling of Macroalgae Species *Ulva ohnoi* and *Oedogonium intermedium*. Clean. Technol..

[99] Viswanathan T., Mani S., Das K.C., Chinnasamy S., Bhatnagar A. (2011). Drying Characteristics of a Microalgae Consortium Developed for Biofuels Production. Trans. ASABE.

[100] Nwakuba N.R., Asoegwu S.N., Nwaigwe K.N. (2016). Energy Requirements for Drying of Sliced Agricultural Products: A Review. Agric. Eng. Int. CIGR J..

[101] Kaveh M., Abbaspour-Gilandeh Y. (2022). Drying Characteristics, Specific Energy Consumption, Qualitative Properties, Total Phenol Compounds, and Antioxidant Activity During Hybrid Hot Air-Microwave-Rotary Drum Drying of Green Pea. Iran. J. Chem. Chem. Eng..

[102] Pradana G.B., Prabowo K.B., Hastuti R.P., Djaeni M., Prasetyaningrum A. (2019). Seaweed Drying Process Using Tray Dryer with Dehumidified Air System to Increase Efficiency of Energy and Quality Product. IOP Conf. Ser. Earth Environ. Sci..

[103] Golpour I., Guiné R.P.F., Poncet S., Golpour H., Amiri Chayjan R., Amiri Parian J. (2021). Evaluating the Heat and Mass Transfer Effective Coefficients during the Convective Drying Process of Paddy (*Oryza sativa* L.). J. Food Process Eng..

[104] Jimenez-Lopez C., Pereira A.G., Lourenço-Lopes C., Garcia-Oliveira P., Cassani L., Fraga-Corral M., Prieto M.A., Simal-Gandara J. (2021). Main Bioactive Phenolic Compounds in *Marine algae* and Their Mechanisms of Action Supporting Potential Health Benefits. Food Chem..

[105] Qu W., Pan Z., Ma H. (2010). Extraction Modeling and Activities of Antioxidants from Pomegranate Marc. J. Food Eng..

[106] Connan S., Delisle F., Deslandes E., Ar Gall E. (2006). Intra-Thallus Phlorotannin Content and Antioxidant Activity in Phaeophyceae of Temperate Waters. Bot. Mar..

[107] Lomartire S., Cotas J., Pacheco D., Marques J.C., Pereira L., Gonçalves A.M.M. (2021). Environmental Impact on Seaweed Phenolic Production and Activity: An Important Step for Compound Exploitation. Mar. Drugs.

[108] Torres P., Osaki S., Silveira E., dos Santos D.Y.A.C., Chow F. (2024). Comprehensive Evaluation of Folin-Ciocalteu Assay for Total Phenolic Quantification in Algae (*Chlorophyta*, *Phaeophyceae*, and *Rhodophyta*). Algal Res..

[109] Dang T.T., Van Vuong Q., Schreider M.J., Bowyer M.C., Altena I.A.V., Scarlett C.J. (2017). The Effects of Drying on Physico-Chemical Properties and Antioxidant Capacity of the Brown Alga (*Hormosira banksii* (Turner) *decaisne*). J. Food Process Preserv..

[110] Sobuj M.K.A., Islam M.A., Islam M.S., Islam M.M., Mahmud Y., Rafiquzzaman S.M. (2021). Effect of Solvents on Bioactive Compounds and Antioxidant Activity of *Padina tetrastromatica* and *Gracilaria tenuistipitata* Seaweeds Collected from Bangladesh. Sci. Rep..

[111] Mekinić I.G., Skroza D., Šimat V., Hamed I., Čagalj M., Perković Z.P. (2019). Phenolic Content of Brown Algae (*Pheophyceae*) Species: Extraction, Identification, and Quantification. Biomolecules.

[112] 112. Kanda H., Kamo Y., Machmudah S., Wahyudiono, Goto M. (2014). Extraction of Fucoxanthin from Raw Macroalgae Excluding Drying and Cell Wall Disruption by Liquefied Dimethyl Ether. Mar. Drugs.

[113] Maeda H., Fukuda S., Izumi H., Saga N. (2018). Anti-Oxidant and Fucoxanthin Contents of Brown Alga Ishimozuku (*Sphaerotrichia divaricata*) from the West Coast of Aomori, Japan. Mar. Drugs.

[114] Savira A.D., Amin M.N., Alamsjah M.A. (2021). The Effect of Different Type of Solvents on the Antioxidant Activity of Fucoxanthin Extract from Brown Seaweed *Sargassum duplicatum*. IOP Conf. Ser. Earth Environ. Sci..

[115] Xia S., Wang K., Wan L., Li A., Hu Q., Zhang C. (2013). Production, Characterization, and Antioxidant Activity of Fucoxanthin from the Marine Diatom *Odontella aurita*. Mar. Drugs.

[116] Nie J., Chen D., Lu Y., Dai Z. (2021). Effects of Various Blanching Methods on Fucoxanthin Degradation Kinetics, Antioxidant Activity, Pigment Composition, and Sensory Quality of *Sargassum fusiforme*. LWT.

[117] Priecina L., Karklina D., Kince T. (2018). The Impact of Steam-Blanching and Dehydration on Phenolic, Organic Acid Composition, and Total Carotenoids in Celery Roots. Innov. Food Sci. Emerg. Technol..

[118] Di Valentin M., Meneghin E., Orian L., Polimeno A., Büchel C., Salvadori E., Kay C.W.M., Carbonera D. (2013). Triplet-Triplet Energy Transfer in Fucoxanthin-Chlorophyll Protein from Diatom *Cyclotella meneghiniana*: Insights into the Structure of the Complex. Biochim. Biophys. Acta Bioenerg..

[119] Wang J., Yang X.H., Mujumdar A.S., Fang X.M., Zhang Q., Zheng Z.A., Gao Z.J., Xiao H.W. (2018). Effects of High-Humidity Hot Air Impingement Blanching (HHAIB) Pretreatment on the Change of Antioxidant Capacity, the Degradation Kinetics of Red Pigment, Ascorbic Acid in Dehydrated Red Peppers during Storage. Food Chem..

[120] Adarshan S., Sree V.S.S., Muthuramalingam P., Nambiar K.S., Sevanan M., Satish L., Venkidasamy B., Jeelani P.G., Shin H. (2024). Understanding Macroalgae: A Comprehensive Exploration of Nutraceutical, Pharmaceutical, and Omics Dimensions. Plants.

[121] Dong J., Li S., Zhang J., Liu A., Ren J. (2022). Thermal Degradation of Cyanidin-3-O-Glucoside: Mechanism and Toxicity of Products. Food Chem..

[122] Ummat V., Sivagnanam S.P., Rai D.K., O’Donnell C., Conway G.E., Heffernan S.M., Fitzpatrick S., Lyons H., Curtin J., Tiwari B.K. (2024). Conventional Extraction of Fucoidan from Irish Brown Seaweed *Fucus vesiculosus* Followed by Ultrasound-Assisted Depolymerization. Sci. Rep..

[123] Nunes A., Dutra F.d.S., Brito S.d.N.S., Pereira-Vasques M.S., Azevedo G.Z., Schneider A.R., Oliveira E.R., dos Santos A.A., Maraschin M., Vianello F. (2024). Effect of Biomass Drying Protocols on Bioactive Compounds and Antioxidant and Enzymatic Activities of Red Macroalga *Kappaphycus alvarezii*. Methods Protoc..

[124] Baek S.H., Cao L., Jeong S.J., Kim H.R., Nam T.J., Lee S.G. (2021). The Comparison of Total Phenolics, Total Antioxidant, and Anti-Tyrosinase Activities of Korean Sargassum Species. J. Food Qual..

